# The Quality of Life of Older Individuals Following the World Health Organization Assessment Criteria

**DOI:** 10.3390/geriatrics5040102

**Published:** 2020-12-05

**Authors:** Margarida Goes, Manuel José Lopes, João Marôco, Henrique Oliveira, César Fonseca, Lisete Mónico, Pedro Parreira, José García-Alonso, Lara Guedes de Pinho

**Affiliations:** 1 Higher School of Health, Polytechnic Institute of Beja, 7800-295 Beja, Portugal; margarida.goes@ipbeja.pt; 2 University of Évora, Comprehensive Health Research Center (CHRC), 7000-811 Évora, Portugal; mjl@uevora.pt (M.J.L.); cfonseca@uevora.pt (C.F.); lmgp@uevora.pt (L.G.d.P.); 3 University Institute of Psychological, Social and Life Sciences, 1149-041 Lisbon, Portugal; joao.maroco@ispa.pt; 4 Telecommunications Institute, Instituto Superior Técnico, 1049-001 Lisbon, Portugal; 5 Faculty of Psychology of University of Coimbra, University of Coimbra, 3000-115 Coimbra, Portugal; lisete.monico@fpce.uc.pt; 6 Health Sciences Research Unit: Nursing (UICISA:E), Nursing School of Coimbra (ESEnfC), 3000-232 Coimbra, Portugal; parreira@esenfc.pt; 7 University of Extremadura, Centro Universitario de Mérida, 06800 Mérida, Spain; jgaralo@unex.es

**Keywords:** elderly, quality of life assessment, WHOQOL-BREF(PT), confirmatory factor analysis

## Abstract

The aim of this study was to evaluate the psychometric qualities of the WHOQOL-BREF(PT) (the questionnaire developed by the World Health Organization Quality of Life Grpup for quality of life assessment), when applied to Portuguese elderly people residing in a community setting. The psychometric qualities were assessed by confirmatory factor analysis. A hierarchical second-order model and a third model were performed, and all three models presented similar and reasonable adjustment indexes. The data analysis showed that the construct failed only regarding discriminant validity because the correlations between the first-order factors were higher, associated with lower values of average variance extracted. The psychometric qualities found in the original translation/validation of the WHOQOL-BREF(PT) were compared with those found in this study; this study found higher correlations between domains but a similar level of factor reliability. The findings of this study lead to three recommendations: (i) to compute each factor score for each participant using the factor score weights obtained from confirmatory analysis models instead of adopting a unitary weight for each item, as proposed by the authors of the original translation/validation of the WHOQOL-BREF(PT); (ii) to compute a QOL score, which is not included in the original translation/validation; and (iii) to analyze differences between individual scores for each participants, which should be done by a group of health experts.

## 1. Introduction

The strong worldwide interest in the quality of life (QOL) concept and the heterogeneity of its definition led the WHO, through the World Health Organization Quality of Life group (WHOQOL group), to develop the most comprehensive definition of QOL in the scientific literature [1]. This group defines QOL as “a state of complete physical, mental and social well-being and not merely the absence of disease…” [2]. This concept favors a transcultural, multidimensional, subjective view of QOL (self-evaluation) and considers correlating factors such as physical and mental health; independence; social relations; personal convictions and beliefs; and the environment [3].

As a result of the work undertaken by the WHOQOL group, the international WHOQOL-BREF questionnaire [4] was developed for QOL assessment. The international WHOQOL-BREF is a shortened version of the WHOQOL-100 [5]), which has been translated/validated in many countries, including Portugal. The WHOQOL-BREF(PT) (the European Portuguese version of the international WHOQOL-BREF) preserves the WHOQOL-100’s 24 facets and the General Health Facet (GHF); it is cross-cultural and thus can be applied to individuals living in different contexts [6].

The WHOQOL-BREF(PT) is generic and includes the domains of Physical Health (seven items), Psychological (six items), Social Relationships (three items), and the Environment (eight items) as well as the GHF (which includes two general items: how respondents rate their QOL and how they rate their satisfaction with their health) [7].

In translating/validation the WHOQOL-BREF(PT), a team of Portuguese researchers belonging to the WHOQOL group adopted two samples from the entire Portuguese population. Both samples included people aged 18 years and over [7]. The healthy sample included 315 citizens who had no chronic disease and did not take any type of medication, and the clinical sample included 289 citizens with different medical backgrounds coming from three public health units in Coimbra City.

Although the psychometric properties of the WHOQOL-BREF(PT) were studied for the Portuguese population in [7], the elderly population was poorly represented in the sample. Since the aging and QOL of older people is a concern, we reevaluate the psychometric properties of the WHOQOL-BREF(PT) exclusively for the elderly population. We use a sample of 351 people aged 65 years or over residing in a community setting (in their homes or in family members’ or friends’ homes) in the Baixo Alentejo Region (BAR). This Portuguese region was chosen because (i) it faces a delicate and worrying situation of population aging with heterogeneous sociodemographics; (ii) it is a rural area located in the mid-southern part of Portugal’s main territory; (iii) it has very low population density, as geographic distances between villages range from 25 to 120 km; and (iv) the public transportation network is scarce, which presents difficulties in the mobility of older people using their own means. Finally, we compare the psychometric properties/qualities we obtain with those observed by Canavarro et al. [7], which represents the publication of all the research work developed by the Portuguese team of researchers belonging to the WHOQOL group that translated and validated the international WHOQOL-BREF for use in Portuguese language.

## 2. Materials and Methods

### 2.1. Subjects

The Health Ethics Committee of Unidade Local de Saúde do Baixo Alentejo (HECULSBA) [8] was the institutional review board that approved the study protocol, including the study design and how it was performed, how the interviews were conducted and how informed consent was presented to each participant. Moreover, all methods of our research were in accordance with all statements included in the operating regulations of HECULSBA [9], whose document was developed under the Helsinki Declaration with the aim to protect the dignity, privacy and freedom of participants [10]. This study was approved by the HECULSBA [9] on 6 July 2014, with a written decision in the minutes of the meeting with reference number 2/2014, by the board of directors of the same ethics committee.

This research involved the population aged 65 years or older registered in the Unidade Local de Saúde do Baixo Alentejo (ULSBA) elderly database (32,893 citizens) [11]. The sample size was calculated adopting the formulae proposed by Scheaffer et al. [12], stratified by gender (male and female) and age group (65 to 74, 75 to 84, and 85 years or older) and assuming the Neyman optimal allocation. The calculated sample size was 468 elderly individuals randomly selected from the ULSBA elderly database. The inclusion criteria were as follows: (i) individuals aged 65 or older; (ii) individuals interested in participating in the study; (iii) individuals residing in the BAR in their own home or in the home of a family member or friend; and (iv) individuals who were able to make their own decisions in the event of illness or hospitalization due to acute and short-term health care needs. The final sample comprised 351 elderly individuals who signed the informed consent form and answered the WHOQOL-BREF(PT) fully and validly. The biological and sociodemographic characteristics of the sample are listed in Table 1.

### 2.2. Statistical Procedures

**Figure 1:**
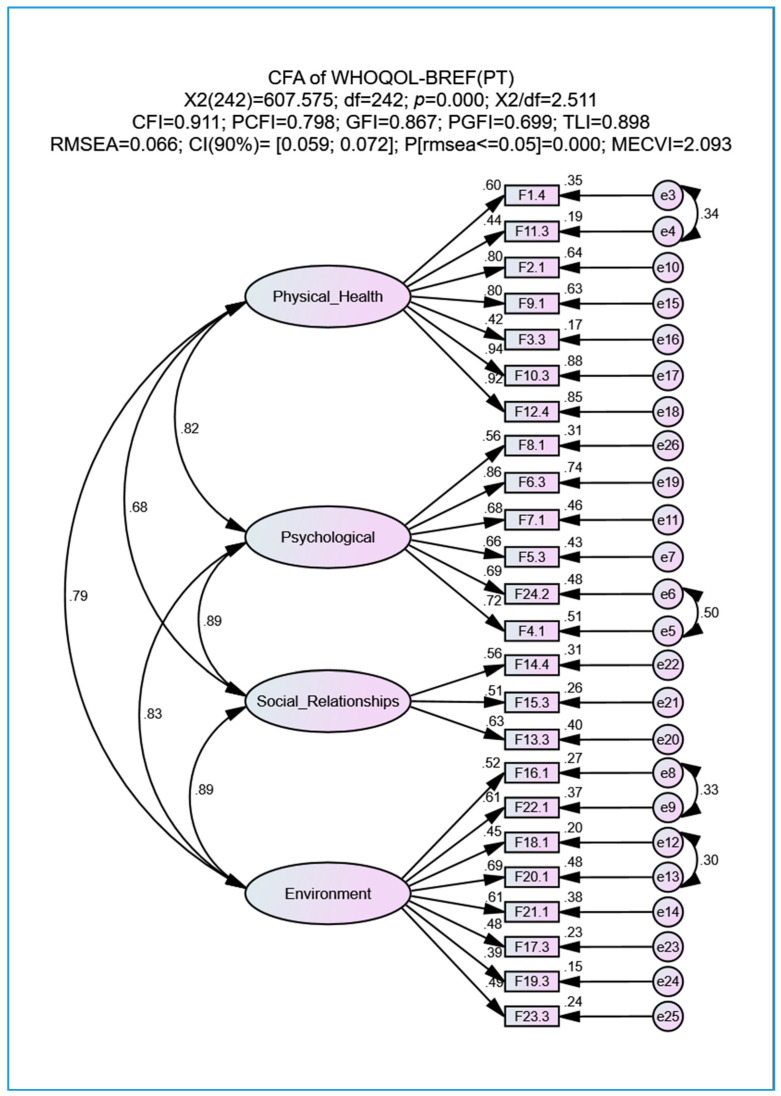
Factorial model of the WHOQOL-BREF(PT) questionnaire after correlating the measurement errors of facets whose modification indexes (MI) suggested a correlation (adopted MI > 11). All abbreviations are defined in the List of Abbreviations section.

Data were collected between January 2016 and April 2017 at citizens’ homes by teams of ULSBA health professionals using a structured interview methodology after prior authorization was given by the authors of the WHOQOL-BREF(PT) [6]. Each item of the WHOQOL-BREF(PT) matches a specific facet of the WHOQOL-100 [5] (see Figure 1, with small rectangles labeled with the letter “F...”, i.e., the observed variables).

All WHOQOL-BREF(PT) facets (items) were measured using a five-point Likert scale, with F1.4, F11.3 and F8.1 measured with an inverted scale. The factorial validity of the WHOQOL-BREF(PT) was evaluated by confirmatory factor analysis (CFA) using AMOS software (v.24, SPSS, an IBM company, Chicago, IL, USA) as described in Marôco [13]. The quality adjustment and reliability of the factorial model were verified. The normality assumption was tested through the analysis of skewness (Sk) and kurtosis (Ku) based on the maximum thresholds: |Sk| < 3 and |Ku| < 10. The quality of the overall model fit was assessed using the following adjustment indexes.: (i) chi-squared/degrees of freedom (χ2/df); (ii) comparative fit index (CFI); (iii) parsimonious CFI (PCFI); (iv) goodness of fit index (GFI); (v) parsimonious GFI (PGFI); (vi) Tucker–Lewis Index (TLI); (vii) root mean square error of approximation (RMSEA); (viii) confidence interval of RMSEA at 90% (CI(90%)); (ix) a test of the null hypothesis that the population RMSEA is no greater than 0.05 (PClose); and (x) modified expected cross-validation index (MECVI) to identify the best-fitting model. All of these indexes are reported in Marôco [13].

The items’ individual reliability was measured by their respective standardized factor loadings (λ). Construct reliability (internal consistency) was evaluated with Cronbach’s alpha (α) and an alternative measure, i.e., the CR, computed from the reflective items of each latent factor. Construct validity is determined by verifying whether the items effectively capture the “big picture” being examined by the specific latent factor (factorial validity). Subsequently, convergent validity was evaluated by determining the average variance extracted (AVE) of each latent factor (usually AVE ≥ 0.5). Discriminant validity was assessed by the positive validity of the expression ((AVE_i_ > ρ_ij_^2)∧(AVE_j_ > ρ_ij_^2)), where *ij* represents two latent factors and ρ_ij_^2 is the square of their correlation. The model adjustments were performed based on MI values (MI > 11; *p* < 0.001). All of these tasks are reported in Marôco [13].

## 3. Results

The 24 facets show no substantial violation of normality because |Sk| ≤ 0.934 and |Ku| ≤ 1.301. The initial CFA model shows a poor overall quality of fit (see Table 2). To obtain a better model fit, the measurement errors between certain facets were correlated, as suggested by MI values, with the assumption that all respective items involved presented some similar content. After all possible adjustments were made to the initial model, we obtained the best-fitting model (a lower MECVI = 2.093; values presented at the top of Figure 1 and Table 2). Regarding the standardized factor loadings (0.391 ≤ *λ* ≤ 0.938, *p* < 0.001 and average = 0.626), approximately 75% present values *λ* ≥ 0.5, 13% present values between 0.5 and 0.45 (near 0.5), and only 12% present values lower than 0.45 (the lowest *λ* = 0.391 for item F19.3). According to Canavarro et al. [7], values of *λ* ≥ 0.3 are admissible for this type of construct. With respect to FC, only the Social Relationships domain (CR_Social Relationships_ = 0.590) presents a value lower than 0.7 (with CR ≥ 0.70 recommended as a positive threshold for CR in Marôco [13]): (i) CR_Physical Health_ = 0.880; (ii) CR_Psychological_ = 0.849; and (iii) CR_Environment_ = 0.761. In the reliability analysis based on the α value, the Social Relationships domain presents the lowest value (α_Social Relationships_ = 0.580), with the remaining domains presenting 0.774 ≤ α ≤ 0.876. Factorial validity is guaranteed since the items (facets) are aligned with what each specific latent factor measures. With respect to convergent validity, only the Physical Health domain presents an acceptable value of AVE_Physical Health_ = 0.532. We observe a nearly acceptable value of AVE_Psychological_ = 0.488, a weak value of AVE_Social Relationships_ = 0.326 and a very weak value of AVE_Environment_ = 0.291 (with AVE ≥ 0.50 recommended as a positive threshold for AVE in Marôco [13] in the case of exploratory investigations). Finally, the adjusted model presents no discriminant validity since the results of all possible expressions (AVE_i_>ρ_ij_^2)∧(AVE_j_>ρ_ij_^2)) are false.

**Figure 2:**
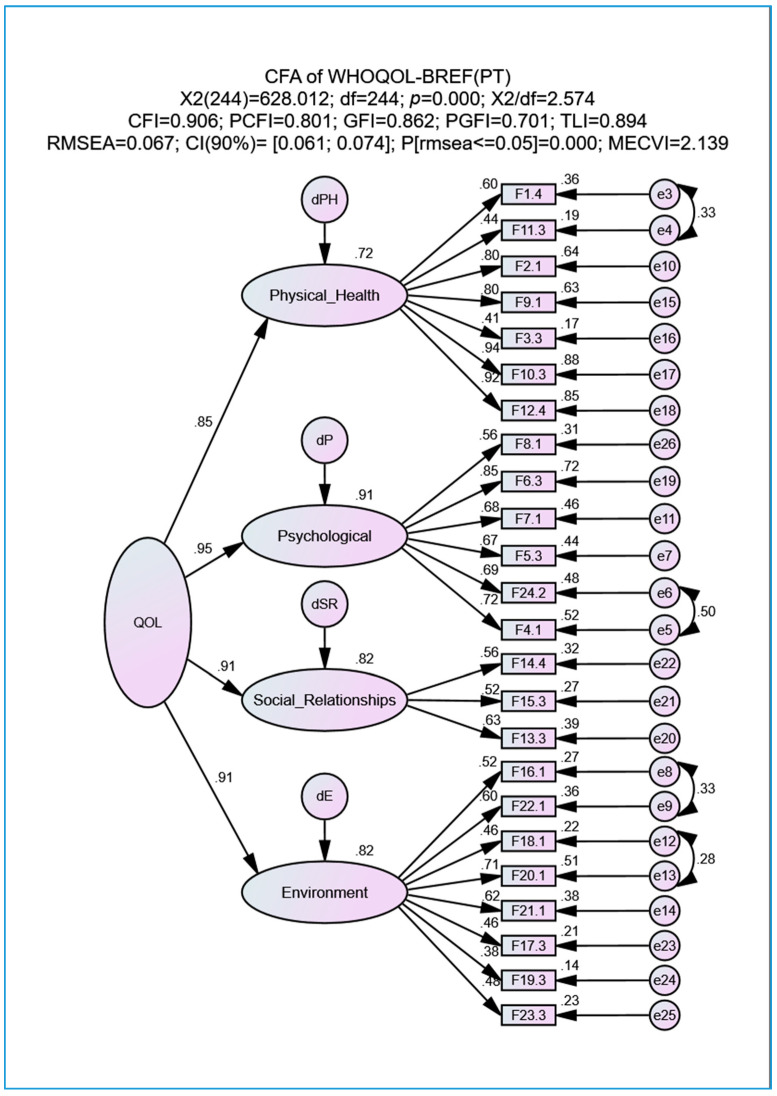
Second-order factorial model (QOL) of the WHOQOL-BREF(PT) after correlating the measurement errors of facets whose MI suggested a correlation (adopted MI > 11). All abbreviations are defined in the List of Abbreviations section.

As an alternative solution to the high correlations between latent factors shown in the model of Figure 1 (all with *p* < 0.001), we performed a hierarchical model (Figure 2) that included a high-order factor called QOL (second-order factor), as recommended for this type of model in Marôco [13]. The values at the top of Figure 2 indicate that this second-order model shows a reasonable adjustment (similar to that presented at the top of Figure 1), with the second-order factor being the QOL measure expressed through various items (observed variables) and associated with each of the four domains. The correlations between QOL and the four domains are all high and statistically significant (*p* < 0.001): (i) ρ_Psychological_ = 0.95; (ii) ρ = 0.91 for both the Social Relationships and Environment domains; and (iii) ρ_Physical Health_ = 0.85. CR and α for the QOL factor achieve very good values of 0.947 and 0.927, respectively.

**Figure 3:**
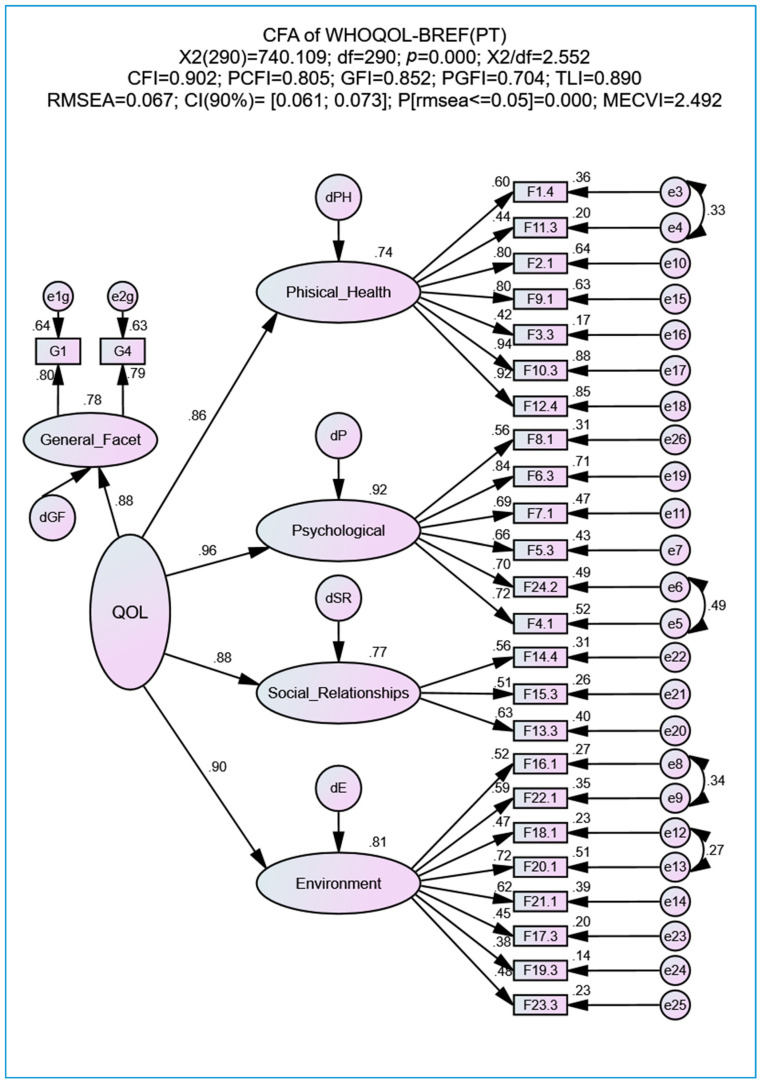
Second-order factorial model (QOL) with the General Health Facet (GHF) of the WHOQOL-BREF(PT) after correlating the measurement errors of facets whose MI suggested a correlation (adopted MI > 11). All abbreviations are defined in the List of Abbreviations section.

The WHOQOL-BREF(PT) includes the GHF, as described in the Background section. We proceeded with a third model (see Figure 3) to verify the correlational effect between the latent factors GHF and QOL. The top part of Figure 3 shows that the adjustment indexes present a similar quality to the second- and first-order models. The correlation between GHF and QOL is high (ρ = 0.88; *p* < 0.001), which allows us to infer that the two factors have concurrent validity.

Table 3 summarizes the descriptive statistics for the four domains, GHF and QOL (24 items), of the WHOQOL-BREF(PT).

## 4. Discussion

The translation and validation performed by the Portuguese team of researchers belonging to the WHOQOL group comprising Canavarro et al. [7] was based on a sample of individuals aged 18 or older. In this paper, a sample of elderly people (65 years and older) was used instead. Therefore, comparisons strictly based on age groups are difficult to perform. However, there are no major differences between the psychometric qualities found in Canavarro et al. [7] and those described here. A large set of psychometric properties were extracted based on the approach developed in this paper, as described below.

The reliability analysis based on Cronbach’s α reported in Canavarro et al. [7] is considered reasonable by the authors. The results in our study are similar to those in that study. The Social Relationships domain presents the lowest value in both studies (α = 0.64 in Canavarro et al. [7] and 0.580 in the present study), and the remaining domains present values ranging from 0.78 ≤ α ≤ 0.86 (in Canavarro et al. [7]) and 0.774 ≤ α ≤ 0.876 (in the present study). In terms of each factor’s reliability, the lowest values for Social Relationships are usually justified by the small number of items included within this domain [3].

Pearson’s correlations reported in Canavarro et al. [7] are all lower than those shown in Figure 1, namely, between the Psychological and Physical Health domains (0.55 and 0.82, respectively), between the Psychological and Social Relationships domains (0.56 and 0.89, respectively), between the Psychological and Environment domains (0.57 and 0.86, respectively), and between the Social Relationships and Environment domains (0.50 and 0.89, respectively). This allows us to state that in our elderly sample, the domains are more interrelated than those in the sample reported by Canavarro et al. [7].

In Canavarro et al. [7], there are no results on the use of a CFA model. Therefore, a comparison with certain results presented in this study cannot be performed. The second-order factor (QOL, see Figure 2) was predicted only by the authors of this paper and not by the authors of the original translation/validation of the questionnaire but only in this paper.

The WHOQOL-BREF(PT) showed factorial validity in the present study (no factor loadings are reported in Canavarro et al. [7]) and presented high correlations between first-order factors but low AVE values. Therefore, the construct failed in discriminant validity because according to Marôco [13], the AVE of the CFA model is less than the square of the correlation between the latent factors involved. Notably, the discriminant validity reported in Canavarro et al. [7] was considered successful. However, discriminant validity in [7] was evaluated as the capability of the WHOQOL-BREF(PT) questionnaire to discriminate between individuals in the normal population and individuals with a medical pathology with respect to all domains and the GHF of QOL. Therefore, the two approaches, i.e., discriminating between sample elements in [7] and extracting domains through CFA in this study, are completely different; therefore, a comparison of the two studies’ results is not possible.

**Figure 4:**
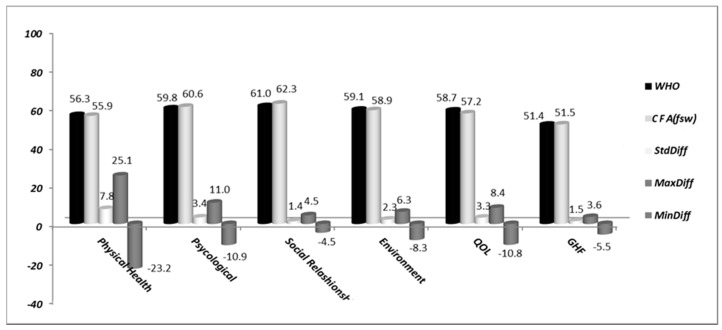
Differences (*Diff*) between average scores of the entire sample based on the WHO strategy and the computations using the extracted *fsw* resulting from CFA. All abbreviations are defined in the List of Abbreviations section.

Based on CFA (a model not developed in [7]), a factor score weight (*fsw*) is extracted for each item and is used to compute each factor score. In the original translation/validation work, each factor score (four domains and GHF) was computed as the average item value (1 to 5) of the items included in the respective domain using a weight value of 1 for each item. However, we propose the computation of factor scores (four domains, GHF and a general QOL) based on the extracted *fsw* (a unique weight for each item). Figure 4 shows the two computation cases, with the average score of the entire sample calculated using the WHO strategy (black cylinders) and using the *fsw* values extracted from CFA (light-gray cylinders). Comparing the six cases, four domains, GHF and QOL generalized score (24 items), there is no significant difference between the two approaches in the global analysis of the sample results. However, analyzing individual scores for each participant separately, we detect differences (maximum positive differences, i.e., *MaxDiff*, and negative differences, i.e., *MinDiff*, both in dark-gray cylinders, as well as the standard deviations of the differences found, i.e., *StdDiff*, in dark-gray cylinders), which are greater for the Physical Health and Psychological domains and weaker than the remaining factor scores.

With respect to the third model (see Figure 3), the correlation between QOL and GHF is strong (ρ = 0.85, *p* < 0.001), suggesting that GHF is a factor that could be used as a QOL generalized measure.

## 5. Conclusions

This paper measures the psychometric qualities of the WHOQOL-BREF(PT) using CFA and applying the questionnaire solely to elderly citizens residing in a rural area with a very low population density. We compare our findings with those of the Portuguese team Canavarro et al. [7]. We believe that our research provides important empirical information that allows a more detailed assessment of the concrete health care needs of elderly individuals who reside in communities with very low population densities (such as in the BAR). The results presented in this research can support the planning and monitoring of health interventions and health policy management and extend the current general knowledge of QOL in the elderly population. No such QOL study has yet been carried out for the elderly population residing in the BAR. With this QOL profile, we expect to determine the factors that are important to the studied population based on the rule of thumb that better knowledge allows better care. The psychometric properties of the WHOQOL-BREF(PT) for the elderly population measured using CFA allow the extraction of *fsw* values, which can help in selecting the most important facets of the WHOQOL-BREF(PT) (those presenting high values of *fsw*) as well as less important facets (those presenting lower values of *fsw*) because these facets contribute differently to the QOL profile. Finally, the *fsw* values can be used as weights to compute new scores of QOL domains, GHF, and QOL (24 items) and compare them to the recommendations of the WHO. The analysis of differences between individual scores for each citizen should be done by health experts.

## Figures and Tables

**Table 1:** Biological and sociodemographic characteristics of the 351 respondents residing in the Baixo Alentejo Region (BAR).

Variables	n	%
*Gender:*		
Male	163	46.4
Female	188	53.6
*Age group:*		
65–74	132	37.6
75–84	135	38.5
85 and higher	84	23.9
*Marital Status:*		
Single/Divorced/Separated	31	8.8
Married/Living as Married	206	58.7
Widowed	114	32.5
*Educational level:*		
Does not know how to read or write	104	29.6
Knows how to read and/or write	59	16.8
1st–4th grade	165	47
More education	23	6.6

**Table 2:** Results from the initial and adjusted confirmatory factor analysis (CFA) models.

Indexes	Initial Model	Adjusted Model	Qualitative Classification Based on Marôco [13]
$\chi^{2}/\mathrm{df}$	3.224	2.511	Good
CFI	0.866	0.911	Good
PCFI	0.772	0.798	Good
GFI	0.832	0.867	Reasonable
PGFI	0.682	0.699	Good
TLI	0.850	0.898	Reasonable
RMSEA	0.080	0.066	Acceptable
CI90%_RMSEA<0.05_	[0.074; 0.086]	[0.059; 0.072]	-
PCLOSE	<0.001	<0.001	-
MECVI	2.598	2.093	Better

**Table 3:** Descriptive statistics of latent variables of the WHOQOL-BREF(PT) questionnaire.

Domains	Mean	Median	SD	Skewness	SE_Sk_	Kurtosis	SE_ku_	Min	Max
Physical Health	3.254	3	1.042	−0.118	0.049	−0.823	0.099	1	5
Psychological	3.392	4	0.901	−0.367	0.053	−0.296	0.107	1	5
Social Relationships	3.439	4	0.842	−0.444	0.075	0.184	0.151	1	5
Environment	3.365	4	0.929	−0.538	0.046	−0.188	0.092	1	5
GHF	3.056	3	0.927	−0.154	0.092	−0.607	0.184	1	5
QOL (24 Items)	3.348	3	0.948	−0.361	0.027	−0.412	0.053	1	5

## List of Abbreviations

WHO: World Health Organization; WHOQOL: World Health Organization Quality of Life; WHOQOL-BREF: The international World Health Organization Quality of Life shortened version questionnaire; WHOQOL-BREF(PT): World Health Organization Quality of Life shortened version questionnaire in Portuguese language; WHOQOL–100: World Health Organization Quality of Life questionnaire with 100 questions; QOL: Quality of Life; GHF: General Health Facet; BAR: Baixo Alentejo Region; HECULSBA: Health Ethics Commission of Unidade Local de Saúde do Baixo Alentejo; ULSBA: Unidade Local de Saúde do Baixo Alentejo; CFA: Confirmatory Factor Analysis; CFI: Comparative Fit Index; PCFI: Parsimonious Comparative Fit Index; GFI: Goodness of Fit Index; PGFI: Parsimonious Goodness of Fit Index; TLI: Tucker Lewis Index; RMSEA: Root Mean Square Error of Approximation; CI (90%): Confidence Interval of Root Mean Square Error of Approximation at 90%; PClose: testing the null hypothesis that the population RMSEA is no greater than 0.05; MECVI: Modified Expected Cross Validation Index; MI: Modification Index; CR: Composite Reliability; AVE: Average Variance Extracted; *fsw*: factor score weights.

## References

[1] Ottati F., Campos M. (2014). Quality of life and coping strategies in the treatment of oncologic patients. Acta Colomb. Psicol..

[2] WHOQOL Group (2020). Introducing the WHOQOL instruments. Measuring Quality of Life. https://www.who.int/toolkits/whoqol.

[3] Fleck M. (2009). A Avaliação da Qualidade de Vida—Guia para Profissionais de Saúde.

[4] WHOQOL Group (2020). Structure of the WHOQOL-BREF. WHOQOL: Measuring Quality of Life. https://www.who.int/toolkits/whoqol/whoqol-bref.

[5] WHOQOL Gorup (2019). The Structure of the WHOQOL-100. WHOQOL: Measuring Quality of Life. https://www.who.int/healthinfo/survey/whoqol-qualityoflife/en/index4.html.

[6] Canavarro M., Serra A., Simões M., Pereira M., Gameiro S., Quartilho M. (2006). WHOQOL-BREF (Versão em Português de Portugal do Instrumento Abreviado de Avaliação da Qualidade de Vida da Organização Mundial de Saúde). http://www.fpce.uc.pt/saude/WHOQOL_Bref.html.

[7] Canavarro M., Serra A., Pereira M., Simões M., Quartilho M., Rijo D., Canavarro M., Serra A. (2010). WHOQOL disponível para Portugal: Desenvolvimento dos Instrumentos de Avaliação da Qualidade de Vida da Organização Mundial de Saúde (WHOQOL-100 e WHOQOL-BREF). Qualidade de Vida e Saúde: Uma Abordagem na Perspectiva da Organização Mundial de Saúde.

[8] HECULSBA (2019). The ULSBA’s Health Ethics Committee (Comissão de Ética para a Saúde da Unidade Local de Saúde do Baixo Alentejo). http://www.ulsba.min-saude.pt/2019/02/28/comissao-de-etica-para-a-saude/.

[9] HECULSBA (2019). The Health Ethics Committee of ULSBA is governed by an Operating Regulation, that was approved on 17 September 2015, by the Board of Directors. http://www.ulsba.min-saude.pt/wp-content/uploads/sites/15/2019/02/Documento-Guia.pdf.

[10] HECULSBA (2019). Helsinki declaration 2008. http://www.ulsba.min-saude.pt/wp-content/uploads/sites/15/2019/02/declaracaohelsinquia.pdf.

[11] ULSBA (2016). Unidade Local de Saúde do Baixo Alentejo. http://www.ulsba.min-saude.pt/.

[12] Scheaffer R., Mendenhall M., Ott R., Gerow K. (2012). Elementary Survey Sampling.

[13] Marôco J. (2014). Análise de Equações Estruturais: Fundamentos teóricos, Software and Aplicações.

